# Endometriosis of the lung: report of a case and literature review

**DOI:** 10.1186/2047-783X-18-13

**Published:** 2013-05-01

**Authors:** Haidong Huang, Chen Li, Paul Zarogoulidis, Kaid Darwiche, Nikolaos Machairiotis, Lixin Yang, Michael Simoff, Eduardo Celis, Tiejun Zhao, Konstantinos Zarogoulidis, Nikolaos Katsikogiannis, Wolfgang Hohenforst-Schmidt, Qiang Li

**Affiliations:** 1 Department of Respiratory Diseases, Changhai Hospital/First Affiliated Hospital of the Second Military Medical University, Shanghai, China; 2 Department of Respiratory Diseases, First Automobile Works General Hospital/The Fourth Affiliated Hospital of Jilin University, Changchun 130021, China; 3 Pulmonary Department, ‘G. Papanikolaou’ General Hospital, Aristotle University of Thessaloniki Medical School, Thessaloniki, Greece; 4 University Pulmonary Department, ‘Ruhrland’ Clinic, University of Duisburg-Essen, Essen, Germany; 5 Surgery Department (NHS), University General Hospital of Alexandroupolis, Alexandroupolis, Greece; 6 Department of Thoracic Surgery, Changhai Hospital/First Affiliated Hospital of the Second Military Medical University, Shanghai, China; 7 Bronchoscopy and Interventional Pulmonology, Pulmonary and Critical Care Medicine, Henry Ford Hospital, Wayne State University, School of Medicine, Detroit, MI, USA; 8 II Medical Clinic, ‘Coburg’ Clinic, University of Würzburg, Coburg, Germany

**Keywords:** Endometriosis, Lung, Pathways

## Abstract

This paper reports a case of endometriosis of the lung in a 29-year-old woman with long-term periodic catamenial hemoptysis. A chest computed tomography image obtained during menstruation revealed a radiographic opaque lesion in the lingular segment of the left superior lobe. During bronchoscopy, bleeding in the mucosa of the distal bronchus of the lingular segment of the left superior lobe was observed. Histopathology subsequent to an exploratory thoracotomy confirmed the diagnosis of endometriosis of the left lung. The 2-year follow-up after lingular lobectomy of the left superior lobe showed no recurrence or complications.

## Background

Endometriosis is the presence of functional endometrial tissue outside the uterus, most commonly in the ovaries, uterosacral ligaments, and peritoneum. It was first described in 1860 [1]. A very common disease in women worldwide, it affects 5% to 15% of them during their reproductive years [2]. Although usually confined to the pelvis, endometriosis is also known to occur in extra-pelvic organs or tissues; ectopic endometrium has been found in the umbilicus, abdominal scars, breasts, the extremities, pleural cavity and lung [3-5]. Although the symptomatology of extra-pelvic endometriosis is always coordinated to the menstrual cycle, this is not directly apparent in all patients and diagnosis is notoriously difficult. Efforts to estimate the prevalence of extra-pelvic endometriosis are hampered by the great variety of symptoms, signs, locations, and ambiguous diagnosis [6].

Endometriosis of the lung is associated with catamenial hemoptysis and chest pain. It is rare, chronic, and estrogen dependent. Confirmation requires a combination of clinical symptoms and postoperative histopathological assessment [7]. Clinical examination often reveals only occult symptoms and signs, but severe cases can result in extensive decidual adhesions and distortion of tissue in the proximity of the decidua. It is these that lead to catamenial pain and hemoptysis, and the disease can be suspected in women with these symptoms [8]. The pathologist’s role is made additionally difficult by the disease’s many histologic features, from typical endometrial glands to an abundance of fibrous tissue.

For women with lung endometriosis, surgery is able to provide radical relief. Because of the high rate of recrudescence, surgical salvage may be expected [9]. Medical therapies have historically included administration of contraceptive progestogens, gonadotropin-releasing hormone agonists, androgens, and non-steroidal anti-inflammatory drugs. Treatments to lower circulating estradiol concentrations may be useful for only a limited time due to unacceptable side effects, and changes or additional medications are commonly needed [10].

Important recent advances in the understanding of endometriosis hold promise for the development of new diagnostic and therapeutic approaches that could limit symptoms and improve fertility [11]. Such new conceptual understandings depend on the study of retrieved endometrial tissue and recognition of the immune response associated with endometriosis.

## Case presentation

A 29-year-old woman complained of repetitive and consecutive catamenial hemoptysis (coughing up of blood during menstruation) of one year’s duration, approximately 5 mL to 10 mL each cycle with blood clots, which gradually had aggravated. Intriguingly, the hemoptysis generally started on the first or second day of each menstruation and thereafter continued for about 6 days, identical with the menstruation interval. The volume of the hemoptysis increased with the menstruation. More importantly, the end of hemoptysis coincided with that of menstruation.

Prior to being seen at our clinic, the patient had taken prescribed antitubercular drugs for 4 months after being misdiagnosed as suffering from pulmonary tuberculosis at the community hospital. Her treatment was ended because hemoptysis had begun again. Hemostasis and fluid administration had been administered during the past 8 months. Hemoptysis had become worse 10 days before, with a hemoptysis volume of 10 mL to 20 mL during each episode accompanied by dyspnea, coincident with the start of menstruation.

**Figure 1:**
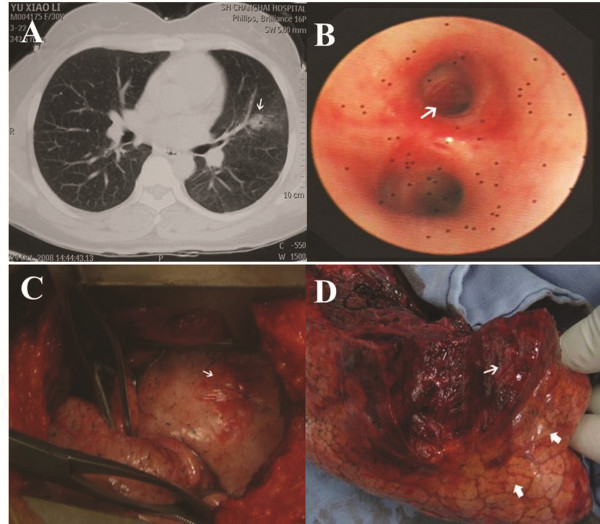
**Clinical findings. (A)** CT scan on the third day of menstruation. There was a nearly round opaque nodule (arrow) in the distal part of the left-superior lobe of the lung. **(B)** Bronchographic image on the fourth day of menstruation. There was bleeding in the distal bronchus of the superior segment of the left lingular lobe. **(C)** Exploration of the lingular lobe of the left lung showed the pleural lamina visceralis was hyperemic and phlyctenular (arrow). **(D)** Making an incision in the left lingular lobe. There was a lesion that constituted the foci of an old hemorrhage (thin arrow) in the pulmonary parenchyma under the pleura. The visceral pleura surrounding the lesion was hyperemic and yellow-staining (thick arrows).

The patient had no history of weight loss or other serious illness. She had achieved conception five times, ending in one normal delivery, one Caesarean section, and three abortions. A physical examination revealed coarse rasps in the left-inferior lobe of the lung with moist rales. A chest radiograph showed irregular nodular opacity in the middle-exterior field of the left lung with a symmetric thorax and plain lung markings. Laboratory findings on admission were as follows: white blood cell (WBC) count was 6,000/μL, creatinine was 1.4 mg/dL, the erythrocyte sedimentation rate (ESR) was 10/h, procalcitonin (PCT) 0.2 ng/mL, C-reactive protein 0.2 mg/dL, AST 38 IU/L, ALT 33 IU/L, LDH 80 IU/L and Hb 8.1 g/dL. A chest computed tomography (CT) image taken during the menstrual cycle and just after a hemoptysis event revealed pneumonia in the left-superior lobe of the lung (Figure 1A). Bronchoscopy (Olympus electric bronchoscope BF-P240 with Number 2800560, Olympus CLV-U40 as the host computer and Olympus CV-240 as the image acquisition system) on the fourth day of the menstrual cycle was performed and showed active bleeding in the distal bronchus of the superior segment of the left lingular lobe (Figure 1B). Bronchoalveolar lavage fluid under the light microscope was negative for acid-fast bacillus. A fibroid space-occupying lesion, 2.2 cm × 1.4 cm, with a clearly defined margin was detectable via pelvic ultrasonography in the anterior wall of the uterus, although gynecological examination showed no signs of abnormalities except for grade-2 erosion of the cervix. Blood cell count and serologic assays did not corroborate the symptoms and signs. Although lung endometriosis was greatly suspected in this patient, she rejected medical treatment for fear of adverse hormonal side effects. Subsequently, this patient suffered a poorer quality of life during one year of catamenial hemoptysis.

A decision to proceed to biopsy was made based on symptoms, signs, and the results of the respiration function test. From the biopsied sample the pathology report confirmed the suspected diagnosis, and a radical resection was subsequently performed. During the exploratory thoracotomy, a phlyctenular apophysis, 2 cm × 2 cm, was detected on the surface of the lingular segment of the left superior lobe with no further macroscopic signs of external endometriosis (Figure 1C). Albuterol 0.83% 5 mL was administered by nebulization twice a day before the surgical procedure. No further hemoptysis was observed at the end of her period after she was admitted. Finally, after the procedure the patient received 5 days of fluid therapy with antibiotic levofloxacin 0.5 (erase 0.5 and add 500 mg) intravenous once a day, as well as sputum therapy with mucosolvan® 90 mg intravenous once a day. The patient was discharged to home on the ninth day in a good condition. The pleura at the site of the lesions’ surfaces was impaired.

### Pathology

#### Gross specimen

The specimen comprised the upper lobe of the left superior lobe, 16.0 cm × 9.0 cm × 4.0 cm. It contained an irregular dark-red lesion, 5 cm × 4 cm × 4 cm, with an obscure boundary 4 cm from the bronchus. The stiffness of the lesion was moderate to mild (Figure 1D).

#### Microscopy

**Figure 2:**
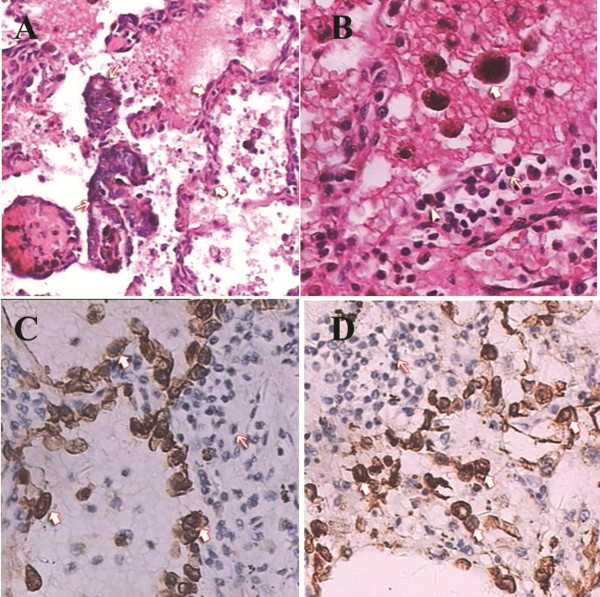
**Staining of pulmonary parenchyma.** H&E: **(A)** Atypical tubular-gland structures of decidual lesions were detected in the alveolar space (thin arrow). Structure of alveolar wall (thick arrow); 100×. **(B)** The alveolar spaces were filled with many red blood cells and phagocytic cells with hemosiderin (thick arrow). The alveolar walls were infiltrated by plasma cells (triangular arrow) and lymphocytes (thin arrow); 200×. Immunohistochemical staining: **(C)** Phagocytic cells in the alveolar space, CD68+ (heavy arrow). Plasma cells and lymphocytes in the alveolar walls, CD68– (thin arrow), H&E 200×. **(D)** Gland epithelium in an alveolus, CK7+ (thick arrow). Infiltrating plasma cells and lymphocytes in the alveolar wall, CK7– (thin arrow), H&E 200×. H&E: hematoxylin and eosin (correct).

Under low-power microscopy, the atypical tubular-gland structures of the lesions were visible in the alveoli and were arrayed as columnar and cubical epithelia, surrounded by a large number of lymphocytes (Figure 2A). In the alveolar space, a large number of red blood cells and phagocytic cells were intermingled. The alveolar walls were infiltrated by plasma cells and lymphocytes (Figure 2B). Immunohistochemical reactions revealed the presence of CD68+ (Figure 2C) and CK7+ (Figure 2D). The postoperative course was uneventful, and the patient was discharged 11 days after surgery. She has been asymptomatic for 2 years without any recurrence of hemoptysis.

## Conclusion

Endometriosis is characterized mainly by the presence of ectopic endometrial glands outside the uterine cavity. It was first reported by Carl von Rokitansky in 1860 [12], who named the condition ‘adenomyoma’. The pathogenesis of endometriosis is ascribed to one (or more) of three mechanisms: the retrograde regurgitation of endometrium through the oviducts during menstruation, Müllerian metaplasia of coelomic epithelium, or lymphatic and venous dissemination of endometrial tissue to distant sites with implantation [11].

Extra-pelvic (that is, non-gynecologic) endometriosis has received special attention primarily because of the diversity of affected sites and its unusual symptomatology. Extra-pelvic endometriosis may occur in any of four anatomical regions: the lungs, bowel-omentum, urinary tract, and all other sites inclusive [13]. Explanations for the spread of endometrial tissues to distant sites rest on hypotheses of venous or lymphatic circulation, or analogies to the metastatic spread of neoplasms [13]. Therefore, the hypothesis is that endometriosis does not result solely from retrograde bleeding, but also from endometrial cells that are shed in the pelvic cavity and which have a tendency to implant and proliferate [14-16].

New insights into the etiology and pathogenesis of this disease serve as a background for new treatments for disease-associated pain and infertility. Possible causal and supportive factors include genetic susceptibility, environmental factors, the immune system, intrinsic endometrial abnormalities, and the secreted products of endometriotic lesions [17]. Importantly, affected women are at higher risk than the general female population of developing ovarian cancer, and may also be at increased risk of breast and other cancers, as well as autoimmune and atopic disorders. However, there may soon be a new repertoire of approaches for treatment and perhaps cure of this enigmatic disorder in the near future.

Endometriosis of the lung is a clinically serious form of the disease, which requires careful differential diagnosis, as in the present case. Our patient complained of hemoptysis, a clinical symptom of thoracic endometriosis, as is hematothorax and pneumothorax. Menstruation-related hemoptysis is not obviously present in all patients, and accurate diagnosis of thoracic endometriosis is always difficult to make. Fortunately, our patient presented with catamenial hemoptysis. Due to our interest in this case, we also reviewed 74 cases of catamenial hemoptysis that have been reported since 1956 [18] in which ectopic endometriosis was identified: 37 cases (59.6%) (please correct to 0.5) were in the right lung, 19 (30.6%) in the left, and 6 (9.7%) were bilateral. In 61 of 70 patients (87.1%) who underwent gynecological examinations, no evidence of pelvic endometriosis was found [1,6-8,18-21]. Additionally, 58 of 73 cases (80.6%) (please correct to 74 and additionally correct the percentage)showed a history of gynecological disorders. These observations support the embolization theory as the underlying cause of ectopic endometriosis in the respiratory tract.

While in our case there was no proven pelvic endometriosis or gynecological disease, our patient has previously undergone induced abortions, and there were two cases in the literature review in which induced abortion may have caused endometriosis in the respiratory system. The CT findings for pulmonary endometriosis may include well-defined opacities, nodular lesions, thin-wall cavities, or bullous formations, but most cases involving hemoptysis have transient radiologic densities in the affected part of the lung [22]. In our patient, the CT images taken during her menstrual period showed a radiographic opacity at the distal end of the left superior lobe. Of note, there was also active bleeding in the distal bronchus of the superior segment of the left lingular lobe detected by bronchoscopy on the fourth day of the menstrual cycle, which further strengthened the clinical diagnosis in the view of the CT finding. Ultimately, histopathological confirmation of ectopic endometriosis was obtained after exploratory thoracotomy.

In general, the initially important diagnostic criterion for this disease is presentation of periodic hemoptysis that is synchronous with menstruation. Most previously reported cases were diagnosed based on the clinical history of the patient; a histological confirmation of ectopic endometriosis is not always done. Fortunately, in the patient reported here, ectopic endometrium was found in the pulmonary parenchyma, and infiltration of hemosiderin-laden macrophages was observed that were the result of repeated bleeding episodes.

Patients with lung endometriosis usually undergo either surgery or hormonal treatments. However, no large-scale randomized trial has been conducted and the optimal treatment regimen remains controversial. Recently, video-assisted thoracic surgery for catamenial hemoptysis was reported, which was found to be safer and less invasive than lobectomy [9,14,23]. When the bleeding site is confirmed by bronchoscopy and it is determined to be distributed within one lobe, the surgical approach is considered appropriate for the disease, especially when the patient desires a pregnancy in the future or if they are worried about possible complications related to hormonal treatments. In addition, the treatment of catamenial hemoptysis by endoscopic laser ablation has also been reported, and this treatment modality should be considered if the lesion can be clearly detected by bronchoscopy [11,13].

In the current case, a lobectomy provided a favorable outcome for this benign lesion, and endometriosis was confirmed via postoperative histopathology. Because of the history of hemoptysis synchronous with the menstrual cycle, an immunohistochemistry test was performed to ensure the preoperative diagnosis. Although we performed a lobectomy rather than the more minimally invasive video-assisted thoracic surgery (because the patient could not afford it), endometriosis-associated events were not present during the postoperative 2-year follow-up. Lobectomy was appropriate for the treatment of lung endometriosis in the present case, and its success lends support for the efficacy and safety of lobectomy for this disorder.

Further studies and a more extensive literature search are required to determine the best course of treatment for patients presenting with this rare disease. Systems biological approaches with patients and morphology-based mathematical modeling could be valuable for studying aspects of pathophysiology and for comparing variants of therapeutic modalities in depth. For the majority of women with endometriosis, the disease imposes a substantial toll in terms of well-being, personal relationships, time away from work, and the need for surgery and expensive therapies [24]. Furthermore, the increased risk of ovarian cancer and possible increased risks of autoimmune diseases and breast and skin cancers further support a need for multidisciplinary care and long-term follow-up of women with this disorder [17].

For women in whom lung endometriosis is suspected, a current history of catamenial hemoptysis, active bleeding observed by bronchoscopy, and evidence from CT scans justify the preliminary diagnosis. The endometriosis patient’s history, environmental exposures, family history, and physical examination are considered just as important for assessment and care. Surgical removal remains the accepted treatment for the disease, and is supported by the outcome of our case. Appropriately designed clinical trials are essential for determining which therapies are safe and effective.

## Consent

Written informed consent was obtained from the patient for publication of this case report and the accompanying images. A copy of the written consent is available for review by the editor-in-chief of this journal

## Abbreviations

CT: computed tomography; H&E: hematoxylin and eosin (correct).

## Competing interests

The authors report no conflicts of interest. The authors alone are responsible for the content and writing of the paper.

## Authors’ contributions

HH and CL made equal contributions to this paper. The two authors diagnosed the patient, treated the patient, and wrote a major part of the manuscript. PZ, NM, NK and KZ were involved in the editing of the manuscript. KD and WH-S assisted as experts in the field. MS and EC revised the manuscript and corrected the final version. LY and TZ treated the patient with HH and CL. QL provided useful insights as an expert. All authors read and approved the final manuscript.
